# Diabetes Modulates MicroRNAs 29b-3p, 29c-3p, 199a-5p and 532-3p Expression in Muscle: Possible Role in GLUT4 and HK2 Repression

**DOI:** 10.3389/fendo.2018.00536

**Published:** 2018-09-12

**Authors:** João V. Esteves, Caio Y. Yonamine, Danilo C. Pinto-Junior, Frederico Gerlinger-Romero, Francisco J. Enguita, Ubiratan F. Machado

**Affiliations:** ^1^Department of Physiology and Biophysics, Institute of Biomedical Sciences, University of São Paulo, São Paulo, Brazil; ^2^Instituto de Medicina Molecular, Faculdade de Medicina, Universidade de Lisboa, Lisbon, Portugal

**Keywords:** microRNA, skeletal muscle, GLUT4, hexokinase, glucose metabolism, glucose uptake, NFKB, streptozotocin

## Abstract

The reduced expression of solute carrier family 2, facilitated glucose transporter member 4 (GLUT4) and hexokinase-2 (HK2) in skeletal muscle participates in insulin resistance of diabetes mellitus (DM). MicroRNAs (miRNAs) have emerged as important modulators of mRNA/protein expression, but their role in DM is unclear. We investigated miRNAs hypothetically involved in GLUT4/HK2 expression in soleus muscle of type 1 diabetes-like rats. *In silico* analysis revealed 651 miRNAs predicted to regulate *solute carrier family 2 member 4* (*Slc2a4*) mRNA, several of them also predicted to regulate *Hk2* mRNA, and 16 miRNAs were selected for quantification. Diabetes reduced *Slc2a4*/GLUT4 and *Hk2*/HK2 expression (50–77%), upregulated miR-29b-3p and miR-29c-3p (50–100%), and downregulated miR-93-5p, miR-150-5p, miR-199a-5p, miR-345-3p, and miR-532-3p (~30%) expression. Besides, GLUT4 and HK2 proteins correlated (*P* < 0.05) negatively with miR-29b-3p and miR-29c-3p and positively with miR-199a-5p and miR-532-3p, suggesting that these miRNAs could be markers of alterations in GLUT4 and HK2 expression. Additionally, diabetes increased the nuclear factor kappa B subunit 1 protein (p50) expression, a repressor of *Slc2a4*, which was also predicted as a target for miR-199a-5p and miR-532-3p. Correlations were also detected between these miRNAs and blood glucose, 24-h glycosuria and plasma fructosamine, and insulin therapy reversed most of the alterations. In sum, we report that diabetes altered miR-29b-3p, miR-29c-3p, miR-199a-5p and miR-532-3p expression in muscle of male rats, where their predicted targets *Slc2a4*/GLUT4 and *Hk2*/HK2 are repressed. These data shed light on these miRNAs as a markers of impaired skeletal muscle glucose disposal, and, consequently, glycemic control in diabetes.

## Introduction

Diabetes mellitus (DM) is a complex metabolic disease characterized by hyperglycemia resulting from defects in insulin secretion and/or action; and type 1 diabetes mellitus (T1DM) and type 2 diabetes mellitus (T2DM) are its major forms. It is known that insulin resistance is a current feature in both T1DM (1) and T2DM (2), and directly contributes to the development and maintenance of glycemic homeostasis loss (3). In this sense, skeletal muscle plays a key role.

The skeletal muscle represents about 40% of body mass; because of that, it is responsible for ~80% of insulin-stimulated glucose uptake in healthy subjects (3), and is considered the major site of peripheral insulin resistance (4). In skeletal muscle and white adipose tissues, the insulin-mediated glucose uptake is performed through the solute carrier family 2, facilitated glucose transporter member 4 (GLUT4) protein (codified by the gene *solute carrier family 2 member 4, SLC2A4*). Part of this protein is restrained in tubule-vesicular structures, which are translocated onto the cell surface in response to the hormone (5, 6). After glucose is transported into the muscular cell, it is phosphorylated by hexokinase-2 (HK2) protein to glucose-6-phosphate and then metabolized (2). Thus, GLUT4 protein is the main mediator of extracellular glucose clearance and HK2 is the key protein in the cellular glucose utilization, both playing a central role in the glycemic homeostasis regulation.

In DM, the reduction of insulin-stimulated glucose uptake and glucose utilization by skeletal muscle is a current feature (3), which has been related to impaired GLUT4 storage vesicles translocation, strongly related to defective glucose transporter gene and/or protein expression (5, 7, 8). In fact, reduced GLUT4 expression in skeletal muscle has been repeatedly observed in different experimental models of diabetes (9–12) as in humans with insulin resistance and T2DM (13–15). Yet, studies with transgenic animals have reinforced the central role of GLUT4 expression in glycemic homeostasis (7). Indeed, muscle-specific deletion of GLUT4 promotes insulin resistance, glucose intolerance and hyperglycemia (16, 17); whereas muscle-specific GLUT4 overexpression in diabetic animals improves insulin sensitivity and glycemic control (18, 19). Hence, reduced GLUT4 expression in skeletal muscle plays a key role in the pathophysiology and treatment of diabetes; and thus, comprehension of mechanisms related to the regulation of *SLC2A4*/GLUT4 expression is mandatory.

In the last years, the epigenetic regulation of genes related to the pathophysiology of diabetes has received great attention, and one of the essential players in this field are the microRNAs (miRNAs). MiRNAs are a class of short (~22 nucleotides) endogenous non-coding RNA molecules that regulate gene expression at the posttranscriptional level, usually through binding at the 3′ UTR region of target mRNAs, destabilizing them or inhibiting their translation (20). It is estimated that over 60% of all mammalian protein-coding genes may be regulated by miRNAs (21). Several miRNAs have been described as involved in the pathophysiology of diabetes and impairment of glycemic homeostasis (22, 23). Some reports have correlated the miRNAs expression with proteins related to the GLUT4 translocation in skeletal muscle in insulin resistance situations (24–26). However, few studies have investigated the regulation of GLUT4 expression by miRNAs, especially in skeletal muscle under conditions of impaired glycemic homeostasis (27). Moreover, the participation of miRNAs in the regulation of HK2 expression in diabetic states is practically unknown.

In this study, we hypothesized that miRNAs participate in the GLUT4 repression in skeletal muscle of diabetic rats, which might be also related to the HK2 expression. Thus, we investigated alterations of expression of miRNAs predicted to regulate *Slc2a4*/GLUT4 and *Hk2*/HK2 expression in skeletal muscle of diabetic rats. Through *in silico* analysis, we have detected that *Slc2a4* and *Hk2* mRNAs are a potential target of hundreds of miRNAs. After evaluating gene, protein and miRNA expression, using correlation analysis, the results show that miR-29b-3p, miR-29c-3p, miR-199a-5p, and miR-532-3p might be candidates to repress GLUT4 and HK2 expression, which could contribute to glycemic homeostasis impairment in diabetes.

## Materials and methods

### Animals and experimental procedures

Fifty-day-old male Wistar rats were obtained from the Animal Center of the Institute of Biomedical Sciences, University of São Paulo. The animals were housed in a room kept at controlled temperature (23 ± 2 C), in 12-h light/12-h dark cycle, with free access to tap water and standard rat chow (Nuvilab CR1; Nuvital Nutrients S/A, Colombo, Paraná, Brazil).

At 70 days of age, after overnight fasting of ~12 h, the animals were rendered diabetic by receiving intravenous injection (dorsal penile vein) of streptozotocin (STZ) (Sigma-Aldrich, St Louis, MO, EUA) at a dose of 50 mg/kg of body weight, solubilized in sodium citrate buffer (0.1 M, pH 4.5); control rats were injected with sodium citrate buffer in the same volume. For this procedure, animals were anesthetized with halothane (Tanohalo®, Cristália, Itapira, SP, Brazil). Thirteen days later, the animals were weighed and kept in metabolic cages during 24 h to evaluate urinary volume, glycosuria and glycaemia; these variables were used to confirm the diabetes condition, and to form the three experimental groups: non-diabetic (ND), diabetic injected with 0.9% NaCl as placebo (D), and diabetic treated with NPH insulin (ID). Insulin treatment was performed twice a day (2 U at 8:00 h and 4 U at 17:00 h; Humulin®, Eli Lilly and Company, Indianapolis); and placebo was injected at the same time. Treatments were conducted for 7 days, totalizing 21 days of diabetes.

At the end of the experimental period, the animals were again kept in metabolic cages for 24-h urine collection. After that, between 8:00 to 10:00 h (after 3–4 h food deprivation), the animals were anesthetized with 50 mg/kg sodium thiopental (Cristália, Itapira, São Paulo, Brazil), tail blood was collected for glucose measurement, and soleus skeletal muscles were gently harvested, weighted and stored at −70°C for further analysis. Additionally, laparotomy was carried out to collect blood from the inferior vena cava to obtain plasma for fructosamine analysis.

All experimental procedures were approved by the Ethical Committee for Animal Research of the Institute of Biomedical Sciences, University of São Paulo (protocol 157/2012), and are in accordance with relevant guidelines and regulations.

### Blood glucose, 24-h urinary glucose excretion, and plasma fructosamine

Blood glucose concentration was measured by a glucometer (Accu-Check Active Basel, Switzerland). Sample from 24-h urine was used to measure glucose concentration by an enzymatic-colorimetric method (Glicose Liquiform, Labtest Diagnóstico S.A., Lagoa Santa, MG, Brazil); the urinary volume was used to calculate the 24-h glucose excretion. Plasma fructosamine concentration was measured by a kinetic-colorimetric method (Frutosamina, Labtest Diagnóstico S.A., Lagoa Santa, MG, Brazil).

### Protein analysis by western blotting

Evaluation of protein expression was performed as previously described (11, 28). Briefly, frozen muscle samples were homogenized in sucrose buffer pH 7.4 (10 mmol/L Tris–HCl, 1 mmol/L EDTA and 250 mmol/L sucrose), centrifuged at 760 g for 10 min at 4°C, and the supernatant was used as a total cellular protein fraction. Protein concentration was determined by Bradford method (Bio-Rad Laboratories, Hercules, CA, USA).

Equal amounts of protein (20–50 μg, according to the target protein) were electrophoresed, transferred to nitrocellulose membrane and immunoblotted with anti-GLUT4 (1:3500, EMD Millipore, Billerica, MA, USA, #07-1404), anti-HK2 (1:1,000, Cell Signaling Technology, Boston, MA, USA, #2867S), anti-NFKB1 (1:1000, Cell Signaling Technology, Boston, MA, USA, #12540S) or anti-RELA (1:450, Abcam, Cambridge, MA, USA, #7970). The membranes were incubated with appropriate secondary conjugated antibody, according to manufacturer's specifications, and signal was detected by enhanced chemiluminescence procedure. The optical density of the blots was quantified by densitometry (ImageScanner III, GE Healthcare, Uppsala, Sweden) and normalized by the densitometry of the respective lane measured in the Ponceau S stained membrane (29). Results were expressed as arbitrary units (AU) per μg of protein, and considering the mean of control values as 100.

### Gene expression analysis by RT-qPCR

Evaluation of mRNA expression was performed by reverse transcription quantitative real-time polymerase chain reaction (RT-qPCR) as previously described (30). Briefly, total RNA from frozen muscle samples were isolated with TRIzol® Reagent (Invitrogen, Carlsbad, CA, USA), according to manufacturer's specifications. Total RNA concentration in each sample was quantified spectrophotometrically (Epoch, BioTek Instruments, Winooski, VT, USA) and RNA purity was estimated by the A_260_/A_280_ ratio. The RNA integrity was checked by the presence of 18S and 28S bands in a 1% denaturant agarose gel electrophoresis exposed to ultra-violet light (Epi Chemi II Darkroom, UVP BioImaging Systems, Upland, California, CA, USA).

To synthetize the cDNA strand from 1 μg of total RNA, the reverse transcriptase reaction was performed using random primers (Invitrogen, Carlsbad, CA, USA), and ImProm-II™ Reverse Transcription System (Promega Corporation, Madison, WI, USA), according to manufacturer's specifications. The conditions of RT reaction were 10 min at 25°C, followed by 60 min at 42°C, and 15 min at 72°C. The qPCR amplification was performed using TaqMan® Gene Expression Assays, TaqMan® Universal Master Mix II with UNG (Applied Biosystems Inc., Foster City, CA, USA), and carried out with StepOne Plus Instrument (Applied Biosystems Inc., Foster City, CA, USA). The PCR conditions were 1 cycle of 2 min at 50°C, 1 cycle of 10 min at 95°C and 40 cycles of 15 s at 95°C and 1 min at 60°C. The target genes evaluated were: solute carrier family 2 member 4 (*Slc2a4*), hexokinase 2 (*Hk2*), nuclear factor kappa B subunit 1 (*Nfkb1*), RELA proto-oncogene and NF-kB subunit (*Rela*); the reference genes were: glyceraldehyde-3-phosphate dehydrogenase (*Gapdh*), actin, beta (*Actb*), hypoxanthine phosphoribosyltransferase 1 (*Hprt1*) and beta-2 microglobulin (*B2m*). The reference gene selected was *B2m*, according to RefFinder algorithm analysis. The method of 2^−ΔΔ*Ct*^ was adopted for analysis of relative levels of mRNA expression (31). The primers used and assay IDs are depicted in Table 1.

**Table 1:** Details for the primers and identification (ID) codes of the TaqMan Gene Expression Assays used for quantitative real-time polymerase chain reaction (qPCR).

**Gene**	**Primer sequence**	**Amplicon length**	**Assay ID**
*Slc2a4*	Fw 5′-GGC TGT GCC ATC TTG ATG AC-3′ Rv 5′-CACGAT GGA CAC ATA ACT CAT GGA T-3′ Probe-5′ FAM AAC CCG CTC CAG CAG C *MGB*3′	75	AI5IQJM 186914021_1
*Hk2*	Inventoried	69	Rn00562457_m1
*Nfkb1*	Inventoried	67	Rn01399572_m1
*Rela*	Inventoried	67	Rn01502266_m1
*Gapdh*	Inventoried	87	Rn99999916_s1
*Actb*	Inventoried	91	Rn00667869_m1
*Hprt1*	Inventoried	64	Rn01527840_m1
*B2m*	Inventoried	58	Rn00560865_m1

### miRNA expression analysis by RT-qPCR

Evaluation of miRNA expression was performed as previously described (32). Briefly, total RNA samples (100 ng) were reverse transcribed using TaqMan® MicroRNA Reverse Transcription Kit and specific miRNA primers from the TaqMan® MicroRNA Assays (Applied Biosystems Inc., Foster City, CA, USA). The RT conditions were: 30 min at 16°C, 30 min at 42°C, 5 min at 85°C, followed by cooling at 4°C. The cDNAs of each miRNA were amplified using TaqMan® 2X Universal PCR Master Mix, No AmpErase® UNG and specific probes for the target miRNAs (Applied Biosystems Inc., Foster City, CA, USA), using a StepOne Plus Instrument (Applied Biosystems Inc., Foster City, CA, USA). The PCR conditions were 1 cycle of 10 min at 95°C, and 45 cycles of 15 s at 95°C and 1 min at 60°C. Target sequences and assay IDs for miRNAs and reference genes are depicted in Table 2. The reference gene selected was U6 snRNA, according to RefFinder algorithm analysis. The method of 2^−ΔΔ*Ct*^ was adopted for analysis (31).

**Table 2:** Target sequences and Taqman assay IDs of the miRNAs and reference genes.

**MicroRNA**	**Sequence**	**Assay ID**
miR-29a-3p	UAGCACCAUCUGAAAUCGGUUA	002112
miR-29b-3p	UAGCACCAUUUGAAAUCAGUGUU	000413
miR-29c-3p	UAGCACCAUUUGAAAUCGGUUA	000587
miR-31a-5p	AGGCAAGAUGCUGGCAUAGCUG	000185
miR-93-5p	CAAAGUGCUGUUCGUGCAGGUAG	001090
miR-106b-5p	UAAAGUGCUGACAGUGCAGAU	000442
miR-150-5p	UCUCCCAACCCUUGUACCAGUG	000473
miR-186-5p	CAAAGAAUUCUCCUUUUGGGCU	002285
miR-199a-5p	CCCAGUGUUCAGACUACCUGUUC	000498
miR-338-3p	UCCAGCAUCAGUGAUUUUGUUGA	000548
miR-345-3p	CCCUGAACUAGGGGUCUGGAGA	002061
miR-377-3p	UGAAUCACACAAAGGCAACUUUU	465108_mat
miR-532-3p	CCUCCCACACCCAAGGCUUGCA	002355
miR-540-3p	AGGUCAGAGGUCGAUCCUGGGC	461976_mat
miR-673-5p	CUCACAGCUCCGGUCCUUGGAG	002054
miR-874-3p	CUGCCCUGGCCCGAGGGACCGA	002268
U87	ACAATGATGACTTATGTTTTTGCCGTTTACCCAGCTGAGGGTTTCTTTGAAGAGAGAATCTTAAGACTGAGC	001712
4.5S RNA(H)	GCCGGTTGTGGTGGCGCACACCGGTAGGATTTGCTGAAGGAGGCAGAGGCAGGAGGATCACGAGTTCGAGGCCAGCCTGGGCTACACATTT	001716
U6 snRNA	GTGCTCGCTTCGGCAGCACATATACTAAAATTGGAACGATACAGAGAAGATTAGCATGGCCCCTGCGCAAGGATGACACGCAAATTCGTGAAGCGTTCCATATTTT	001973

### *In silico* analysis of miRNAs predict to regulate target genes

The search for miRNAs targeting the mRNAs *Slc2a4, Hk2, Nfkb1* and *Rela* was performed using the miRWalk database 2.0, a comprehensive atlas of predicted and validated miRNA–target interactions (33). The analysis was restricted to the target mRNA 3′-UTR region of rat *Slc2a4, Hk2* and *Rela* genes, and of human *NFKB1* gene, since there is no data referring to rat *Nfkb1* gene in the miRWalk database. The official nomenclature and sequence annotation of miRNAs is based on the “miRBase Sequence Database-Release 21.”

### Statistical analysis

All data were expressed as mean ± standard error mean (SEM). The comparison of the means, according to normality of the data distribution (Shapiro-Wilk test), was performed by one-way analysis of variance (ANOVA) or Kruskal-Wallis test, followed by the Bonferroni or Dunn *post hoc* test, respectively. Correlations between proteins and miRNAs, or metabolic variables and miRNAs, were performed by Pearson (r) or Spearman (ρ) correlation coefficient analysis. Comparisons were considered statistically significant at *p* < 0.05. Analyses were performed using GraphPad Prism 5.0 (GraphPad Software Inc.,).

## Results

### Characteristics of diabetic rats

Table 3 shows that, as expected, diabetic rats reduced body and skeletal muscle mass development, and increased blood glucose concentration, 24-h urinary glucose excretion and plasma fructosamine concentration. Insulin treatment restored all parameters, except plasma glucose concentration, which, despite 20% reduction (*P* < 0.001 vs. D), remained still higher than the ND value (*P* < 0.001).

**Table 3:** Morphometric and metabolic characteristics of non-diabetic (ND), diabetic (D) and insulin-treated diabetic (ID) rats.

	**ND**	**D**	**ID**
Body weight (g)	365.2 ± 10.1	256.3 ± 4.7^***^	315.5 ± 10.1^##^
Soleus muscle weight (mg)	129.0 ± 8.1	90.4 ± 5.9^**^	118.3 ± 7.7^#^
Blood glucose (mg/dL)	104.4 ± 4.0	500.5 ± 13.9^***^	402.4 ± 25.1^***^^###^
Glycosuria (mg/24 h)	3.29 ± 0.9	387.9 ± 35.8^***^	82.1 ± 12.7^###^
Plasma fructosamine (μmol/L)	157.2 ± 8.1	240.1 ± 15.1^***^	188.2 ± 10.2^#^

*Data are mean ± SEM of 6-15 animals. One-way ANOVA followed by Bonferroni post-test or Kruskal-Wallis test followed by Dunn post-test, as suitable*.

** P < 0.01 and

*** *P < 0.001 vs. ND*;

# *P < 0.05*,

## P < 0.01, and

### *P < 0.001 vs. D*.

### Diabetes reduces *Slc2a4*/GLUT4 and Hk2/HK2 expression in skeletal muscle

**Figure 1:**
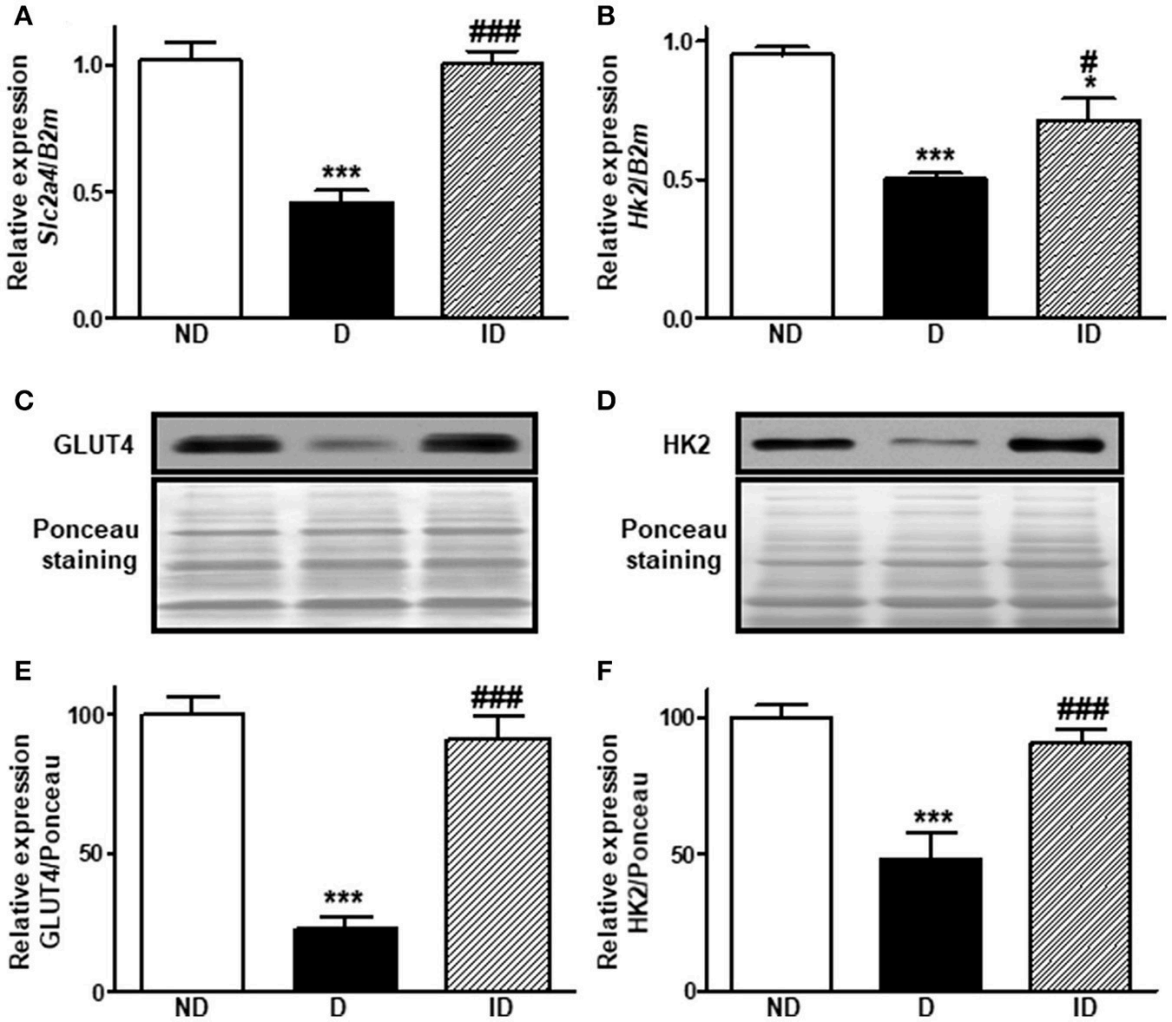
Diabetes reduces Slc2a4/GLUT4 and Hk2/HK2 expression in skeletal muscle. Relative expression of *Slc2a4* **(A)** and *Hk2* **(B)** mRNAs and GLUT4 **(C,E)** and HK2 **(D,F)** proteins in soleus skeletal muscle of non-diabetic (ND), diabetic (D) and insulin-treated diabetic (ID) rats. **(C,D)** Are typical images of immunoblots of GLUT4 and HK2 proteins, and their respective Ponceau-stained membranes. (**E**,**F)** Are densitometry analysis of GLUT4 and HK2 proteins. Data are mean ± SEM of 7–12 animals per group. One-way ANOVA followed by Bonferroni post-test: ^*^P < 0.05 and ^***^P < 0.001 vs. ND; ^#^*P* < 0.05 and ^###^*P* < 0.001 vs. D.

Diabetes reduced (*P* < 0.001) by ~55 and ~77% the *Slc2a4* mRNA (Figure 1A) and GLUT4 protein (Figures 1C,E), respectively. Similarly, *Hk2* mRNA (Figure 1B) and HK2 protein (Figures 1D,F) were reduced (~50%) in diabetic rats. Insulin treatment restored these variables completely, except *Hk2* mRNA, which was only partially reversed (*P* < 0.05 vs. ND and *P* < 0.05 vs. D).

### Hundreds of miRNAs are predicted to regulate *Slc2a4* and/or *Hk2* mRNAs

**Figure 2:**
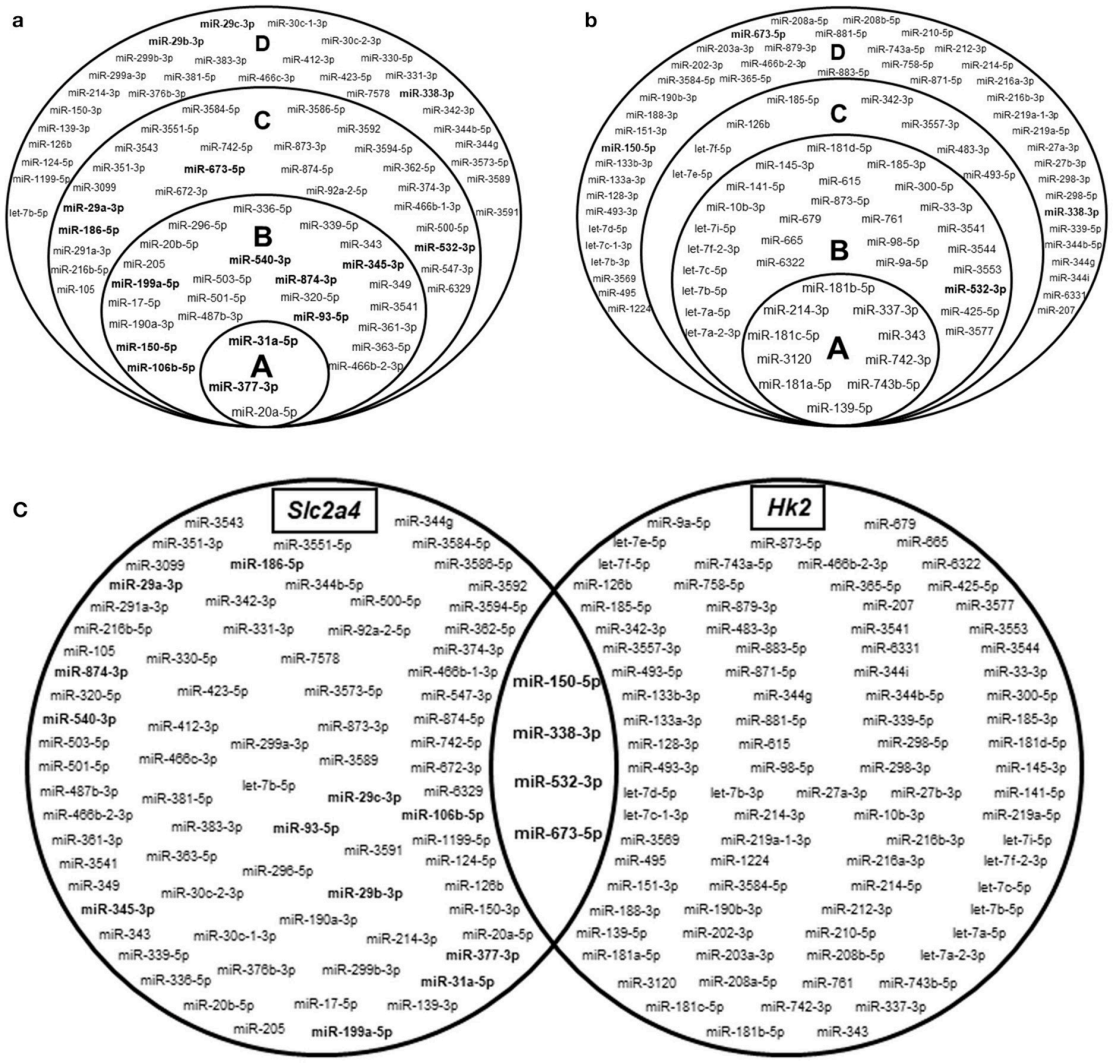
Hundreds of miRNAs are predicted to regulate Slc2a4 and Hk2 mRNAs. Stacked Venn diagrams showing miRNAs candidates to regulate *Slc2a4* and *Hk2* in rat. For *Slc2a4* **(a)**, circles A, B, C, and D indicate miRNAs predicted by 7, 6, 5, and 4 algorithms, respectively. For *Hk2* **(b)**, circles A, B, C, and D indicate miRNAs predicted by 6, 5, 4, and 3 algorithms, respectively. **(c)** Basic Venn diagram shows the merged miRNAs presented in the stacked Venn diagrams. The miRNAs evaluated in this study were highlighted in bold.

*In silico* analysis using the miRWalk 2.0 database revealed that 651 miRNAs were predicted to regulate *Slc2a4* mRNA by at least one algorithm; and stacked Venn diagram (Figure 2a) shows those pointed out by 4–7 algorithms. Similarly, 613 miRNAs were predicted to regulate *Hk2* mRNA and stacked Venn diagram shows those pointed out by 3–6 algorithms (Figure 2b). From these miRNAs, 558 were predicted to regulate both *Slc2a4* and *HK2* mRNAs by at least one algorithm.

Figure 2a highlights the 16 miRNAs selected for analysis in the present study. These miRNAs include: (1) 9 miRNAs (1/3 of 27) predicted to regulate *Slc2a4* mRNA by 6 to 7 algorithms; (2) 4 miRNAs predicted to regulate *Slc2a4* mRNA by less than 6 algorithms, but also predicted to regulate *Hk2* mRNA; (3) 3 miRNAs from the miR-29 family, which are known as regulators of glucose transport and *Slc2a4/*GLUT4 expression in diabetes (34, 35). Figure 2c combines the miRNAs presented in Figures 2a,b, depicting that only 4 miRNAs are highly predicted to regulate both *Slc2a4* and *Hk2* genes.

Selection of 16 miRNAs for quantification was based on their high prediction to regulate *Slc2a4* and previous reports indicate their participation in GLUT4 regulation (7 miRNAs) or in metabolic related dysfunction of muscle and/or adipose cells (9 miRNAs) (34–47).

### Diabetes upregulates miR-29b-3p and miR-29c-3p expression and downregulates miR-93-5p, miR-150-5p, miR-199a-5p, mir-345-3p and Mir-532-3p expression in skeletal muscle

**Figure 3:**
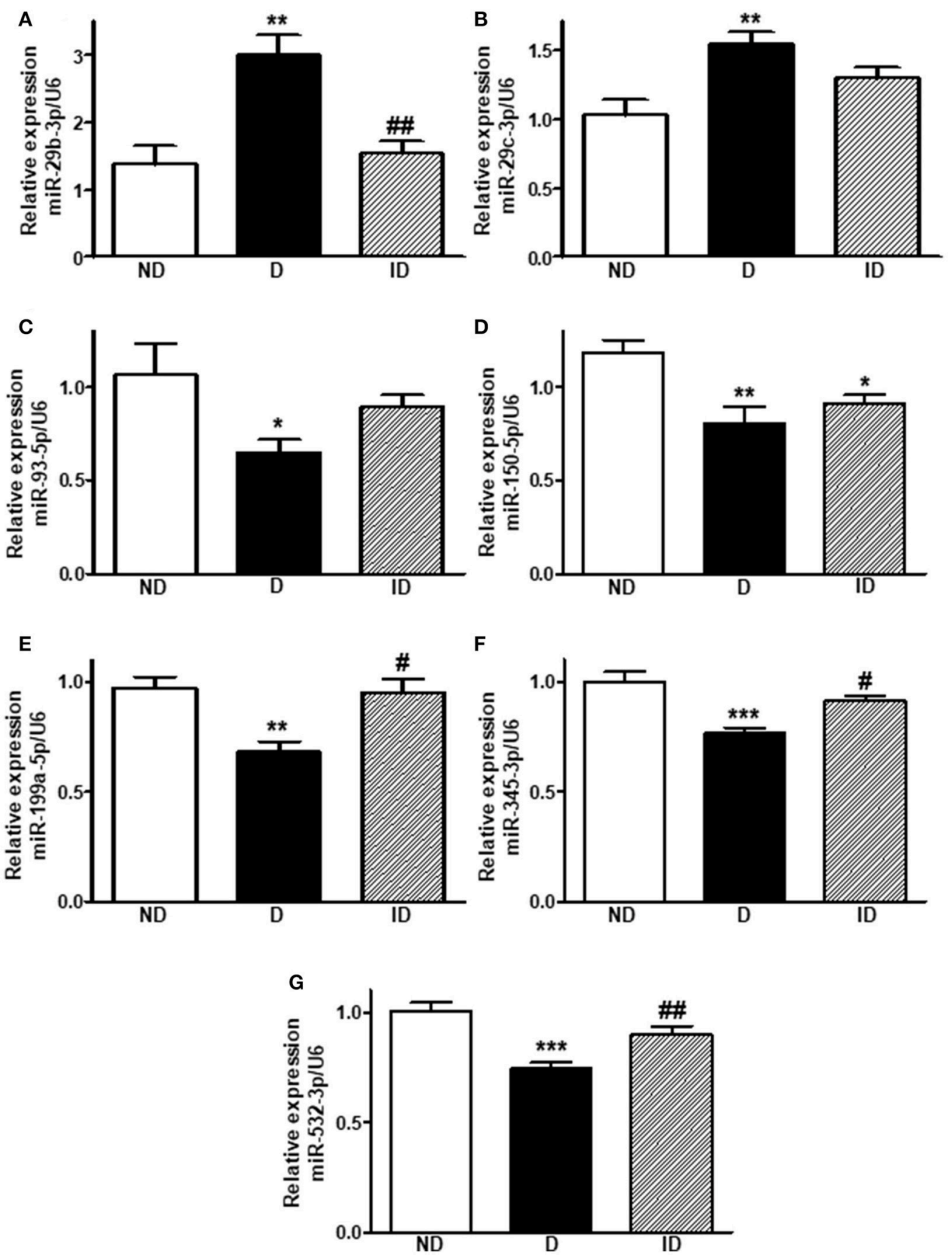
Diabetes upregulates miR-29b-3p and miR-29c-3p expression and downregulates miR-93-5p, miR-150-5p, miR-199a-5p, miR-345-3p, and miR-532-3p expression in skeletal muscle. Relative expression of miR-29b-3p **(A)**, miR-29c-3p **(B)**, miR-93-5p **(C)**, miR-150-5p **(D)**, miR-199a-5p **(E)**, miR-345-3p **(F)**, and miR-532-3p **(G)** in soleus skeletal muscle of non-diabetic (ND), diabetic (D) and insulin-treated diabetic (ID) rats. Data are mean ± SEM of 6–8 animals per group. One-way ANOVA followed by Bonferroni post-test or Kruskal-Wallis followed by Dunn post-test: *P < 0.05, **P < 0.01, and ***P < 0.001 vs. ND; ^#^*P* < 0.05, ^##^*P* < 0.01 vs. D.

Diabetes increased (*P* < 0.01) by ~118 and ~51% the expression of miR-29b-3p (Figure 3A) and miR-29c-3p (Figure 3B), respectively. Insulin treatment completely restored the expression of miR-29b-3p.

On the other hand, five miRNAs were downregulated by diabetes in skeletal muscle (*P* < 0.05 to *P* < 0.001). MiR-93-5p was reduced by ~39% (Figure 3C), miR-150-5p by ~32% (Figure 3D), miR-199a-5p by ~30% (Figure 3E), miR-345-3p by ~23% (Figure 3F), and miR-532-3p was downregulated by ~26% (Figure 3G). Insulin treatment was capable of restoring (*P* < 0.05 to *P* < 0.01) the expression of miR-199a-5p, miR-345-3p and miR-532-3p, but had no effect upon miR-150-5p.

### miR-29b-3p, miR-29c-3p, miR-199a-5p and mIR-532-3p expression correlates with GLUT4, HK2 and glycemic control

Considering the important role of miRNAs upon translation of mRNAs, usually destabilizing and reducing the protein translation, the putative relationship between the altered miRNAs and GLUT4 or HK2 proteins was investigated by correlation analysis (Table 4). Results show that miR-29b-3p and miR-29c-3p expression correlates negatively whereas miR-199a-5p and miR-532-3p expression correlates positively with GLUT4 and HK2 expression. In addition, most of these miRNAs also correlated with metabolic variables (glycemia, glycosuria and/or fructosamine), but in a converse way to that of the proteins, and caveating that the correlation between miR-29c-3p and glycosuria displays a marginal significance (*P* = 0.059) (Table 4).

**Table 4:** Correlation analysis between miRNAs expression and GLUT4, HK2 or metabolic variables.

	**GLUT4 protein**	**HK2 protein**	**Blood glucose**	**24-h glycosuria**	**Plasma fructosamine**
miR-29b-3p	−0.64^***^	−0.41^*^	0.54^**^	0.45^*^	0.44
miR-29c-3p	−0.50^*^	−0.49^*^	0.55^**^	0.41^&^	0.26
miR-93-5p	0.16	0.001	**–**0.18	**–**0.33	**–**0.50
miR-150-5p	0.13	0.13	**–**0.24	**–**0.27	**–**0.24
miR-199a-5p	0.45^*^	0.48^*^	−0.63^***^	−0.56^**^	−0.78^**^
miR-345-3p	0.38	0.03	−0.58^***^	−0.62^**^	−0.81^***^
miR-532-3p	0.54^**^	0.60^**^	−0.54^**^	−0.67^***^	−0.72^**^

*Data are Person (r) or Spearman (ρ) correlation coefficients, according to the normal distribution of the data*.

* *P < 0.05*,

** P < 0.01, and

*** *P < 0.001*,

& *P = 0.059*.

*Bold indicates correlation coefficient statistically significant*.

### NFKB1 is a possible mediator of *Slc2a4*/GLUT4 repression by miR-199a-5p and miR-532-3p

In order to propose how the miR-199a-5p and miR-532-3p, which correlated positively with *Slc2a4*/GLUT4, might be participating in the repression of the *Slc2a4* gene, we searched whether these miRNAs were predicted to target mRNAs from the *Nfkb* family, a classic repressors of *Slc2a4*. *In silico* analysis indicated that miR-199a-5p targets human *NFKB1* and rat *Rela* mRNAs by 7 and 1 algorithms; respectively, whereas miR-532-3p targets only the rat *Rela* mRNA by 2 algorithms. So, the expression of *Nfkb1*/NFKB1 and *Rela*/RELA in skeletal muscle of diabetic rats was evaluated.

**Figure 4:**
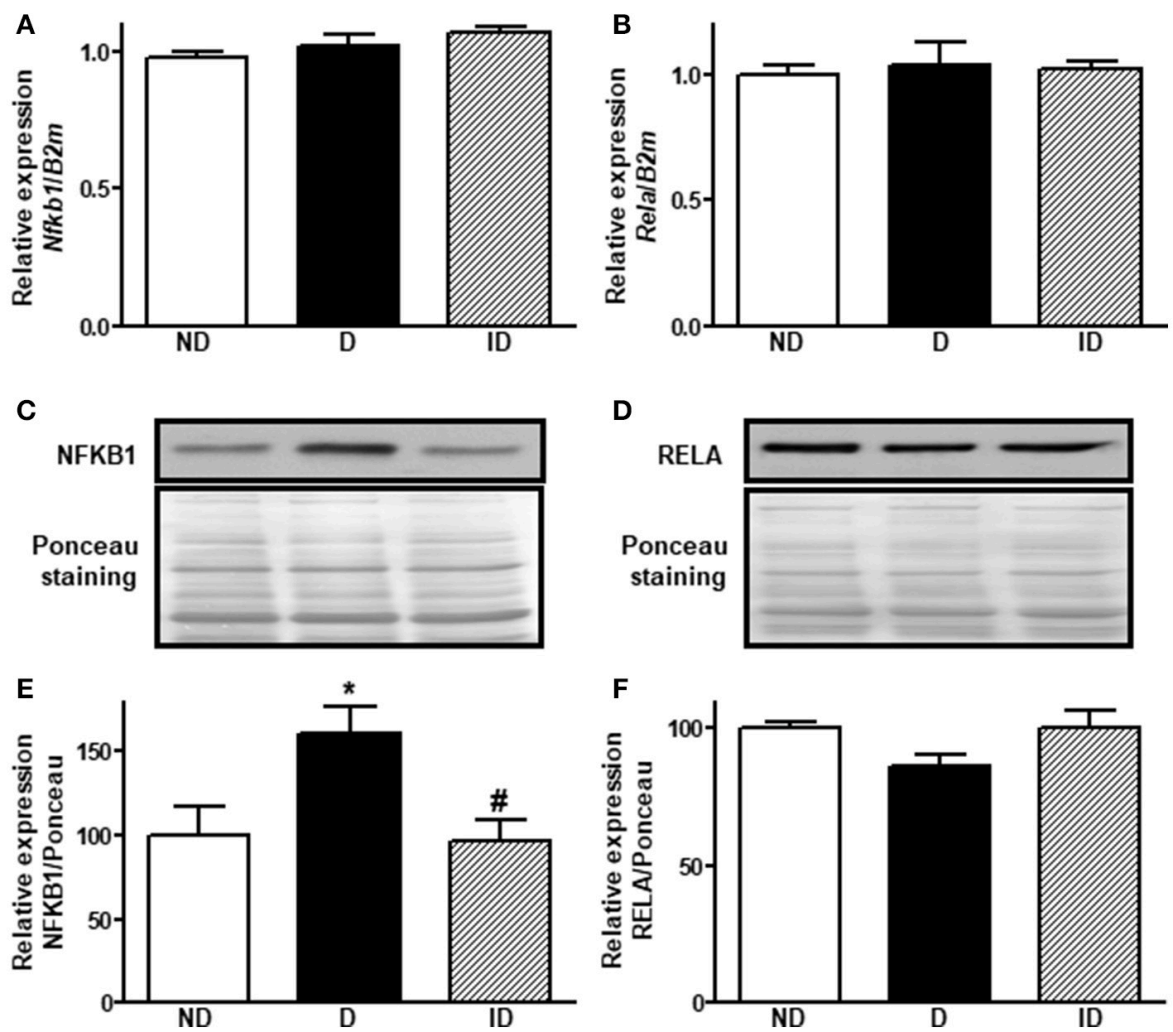
Diabetes induces NFKB1 protein expression in skeletal muscle. Relative expression of *Nfkb1* **(A)** and *Rela* **(B)** mRNAs and NFKB1 **(C,E)** and RELA **(D,F)** proteins in soleus skeletal muscle of non-diabetic (ND), diabetic (D) and insulin-treated diabetic (ID) rats. **(C,D)** Are typical images of immunoblots of NFKB1 and RELA proteins, and their respective Ponceau-stained membranes. **(E,F)** Are densitometry analysis of NFKB1 and RELA proteins. Data are mean ± SEM of 9–11 animals *per* group. One-way ANOVA followed by Bonferroni post-test: *P < 0.05 vs. ND; ^#^*P* < 0.05 vs. D.

Figure 4 shows that diabetes did not alter skeletal muscle *Nfkb1* and *Rela* mRNA expression; however, NFKB1 protein expression (Figures 4C,E) was increased by ~61% (*P* < 0.05), indicating a post-transcriptional modulation. No changes were observed in RELA protein expression.

### Diabetes did not alter miR-29a-3p, miR-31a-5p, miR-106b-5p, miR-186-5p, miR-338-3p, miR-377-3p, miR-540-3p, miR-673-5p and miR-874-3p expression in soleus skeletal muscle

**Figure 5:**
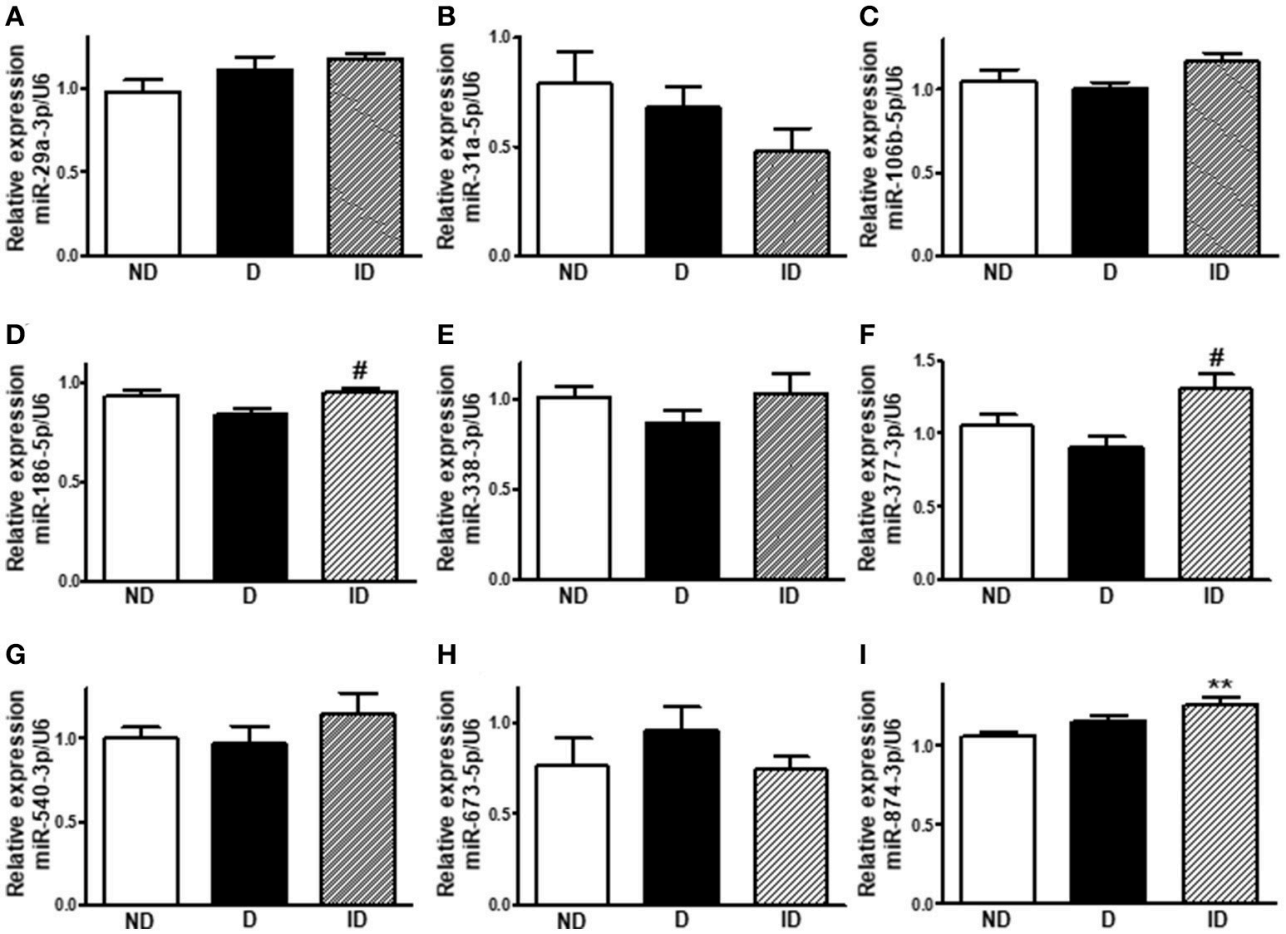
Diabetes did not alter miR-29a-3p, miR-31a-5p, miR-106b-5p, miR-186-5p, miR-338-3p, miR-377-3p, miR-540-3p, miR-673-5p, and miR-874-3p expression in soleus skeletal muscle. Relative expression of miR-29a-3p **(A)**, miR-31a-5p **(B)**, miR-106b-5p **(C)**, miR-186-5p **(D)**, miR-338-3p **(E)**, miR-377-3p **(F)**, miR-540-3p **(G)**, miR-673-5p **(H)**, and miR-874-3p **(I)** in soleus skeletal muscle of non-diabetic (ND), diabetic (D) and insulin-treated diabetic (ID) rats. Data are mean ± SEM of 6–9 animals per group. One-way ANOVA followed by Bonferroni post-test: **P < 0.01 vs. ND; ^#^*P* < 0.05 vs. D.

Figure 5 shows that the expression of 9 out of the 16 miRNAs investigated was not altered in soleus muscle of diabetic-treated mice. However, miR-186-5p and miR-377-3p expression increased in muscle from insulin-treated diabetic rats, as compared to placebo-treated diabetic rats, and miR874-3p expression also increased in insulin-treated rats, as compared to non-diabetic rats.

## Discussion

GLUT4 protein plays a central role in the insulin-regulated blood glucose clearance by skeletal muscle (8, 48). In diabetes, both in T1DM and T2DM, decreased glucose uptake by skeletal muscle participates in the glycemic homeostasis impairment (1, 3, 49), in which reduction in GLUT4 expression must be involved (5, 7, 8, 48). Indeed, reduced GLUT4 expression in skeletal muscle has been repeatedly observed in different experimental models of diabetes (9–11, 12) as in humans with insulin resistance and T2DM (13–15), despite some controversial reports. Thus, by determining defects on *Slc2a4*/GLUT4 expression-related mechanisms, we can contribute to preventive and/or therapeutic approaches for DM (8).

Several studies have inferred modulations in biological phenomena based only on variations in mRNA content, and large-scale analysis of gene expression has contributed very much to that, including studies that involved *Slc2a4* mRNA (50, 51). However, discrepancies between *Slc2a4* mRNA and GLUT4 protein regulations have for long been reported (52, 53), pointing out the imperativeness of correlating function with protein only, and of considering potential posttranscriptional mechanisms involvement in all *Slc2a4*/GLUT4 discrepancies. Indeed, in red muscle from STZ-diabetic rat, nuclear run-on analysis revealed a 35% reduction in *Slc2a4* transcription, whereas *Slc2a4* mRNA was 50% decreased, suggesting a posttranscriptional regulation (54), and discrepancies between *Slc2a4* mRNA and GLUT4 protein regulations have been described in muscle from diabetic and insulin resistant humans and rats (52, 55, 56).

More recently, miRNAs have been extensively implied in posttranscriptional modification of target mRNAs; thus, potentially involved in regulations of *Slc2a4* mRNA and GLUT4 protein (27). Currently, miRNAs have been described as capable of increasing mRNA degradation and/or of inhibiting mRNA translation, the latter inducing discrepancies between mRNA and protein content variations. In this study, DM reduced *Slc2a4*/GLUT4 expression, and reduction in protein (by 77%) was higher than the reduction in mRNA (by 55%), which reinforces the potential participation of miRNAs in these regulations.

Regarding that, among the miRNAs evaluated, only miR-29b-3p and miR-29c-3p were observed to increase in muscle from diabetic rats, and their expression correlates with the GLUT4 expression negatively, suggesting a possible connection, once the miR-29a and−29c have already been validated as repressors of *Slc2a4*/GLUT4 (35). Indeed, predictable regulation of *Slc2a4* mRNA by miR-29a/c-3p has been proposed by *in silico* analysis (57, 58), and the repressor effect of miR-29a-3p was further demonstrated upon GLUT4 expression in C2C12 muscle cells (57). Furthermore, expression of miR-29a-3p and miR-29c-3p, but not of miR-29b-3p, was described as increased in vastus lateralis muscle from patients with T2DM (35), and GLUT4 protein expression was observed to decrease in mice skeletal muscle overexpressing miR-29a-3p and−29c-3p (35). Finally, GLUT4 expression has been reported decreased in 3T3-L1 adipocytes overexpressing miR-29a-3p,−29b-3p or−29c-3p (59), reinforcing the miR-29s/GLUT4 relationship by gain-of-function approach.

On the other hand, despite that only one algorithm predicts *Hk2* mRNA as target for miR-29a/b/c-3p in the *in silico* analysis, the present results reveal that miR-29b-3p and miR-29c-3p expression negatively correlated with the HK2 protein expression. Indeed, miR-29a-3p and miR-29c-3p gain- and loss-of-function approaches in human primary myotubes evinced the repressor effect of these miRNAs upon *HK2* mRNA expression (35), revealing a causal relationship. However, the direct binding of these miRNAs into the *Hk2* mRNA remains to be demonstrated.

Additionally, we highlight that skeletal muscle miR-29 regulation by diabetes seems to vary according to the type of diabetes and muscle fiber. We detected increased miR-29b-3p and 29c-3p in soleus of STZ-diabetic rats; however, Massart et al. (35) reported increased miR-29a- and miR-29c in vastus lateralis of T2DM humans, and increased miR-29a, miR-29b and miR-29c in gastrocnemius of ob/ob mice.

The expression of five miRNAs was detected reduced in soleus muscle from diabetic rats: miR-93-5p, miR-150-5p, miR-199a-5p, miR-345-3p, and miR-532-3p. From those miRNAs, miR-532-3p was proposed to target *Slc2a4* by 5 algorithms, and the other ones by 6 algorithms, making them strong candidates to regulate *Slc2a4*. Furthermore, miR-199a-5p and miR-532-3p displayed a statistically significant positive correlation with GLUT4 and HK2 expression, suggesting a possible connection between these miRNAs and GLUT4 and HK2 expression. Concerning miR-199a, reported to be increased in plasma of T2DM subjects, it was described as capable of repressing GLUT4 (39) and HK2 (60), by means of gain- and loss-of-function approaches, in transfected HEK293T, L6, Huh-7 and/or HepG2 cells. Furthermore, miR-532-3p was confirmed as repressor of HK2, also by gain- and loss-of function, in ovarian cancer cells SKOV3 and A2780 (61). However, in the present study, the expression of these miRNAs is reduced in skeletal muscle of T1DM rats, which, by a direct effect, should contribute to increasing the expression of target tissues, instead of the reduction observed. That means, *in vivo*, in skeletal muscle of T1DM rats, other regulators of gene/protein expression must be overcoming the effect of these miRNAs.

Nevertheless, since miRNAs have been acknowledged as repressors of mRNA stability and/or translation, concomitant decrease in miR-199a-5p, miR-532-3p and their targets, as observed here in muscle, would be incomprehensible. However, some studies have reported that miRNAs have the capability of directly activating target mRNAs (62), which merits future confirmation in the present situation.

Furthermore, these miRNAs, the expression of which was reduced by DM, might be regulating the *Slc2a4*/GLUT4 expression through a repressor mediator. Based on this rationale, we investigate the participation of NFKB, a final mediator of inflammatory pathway, and powerful repressor of *Slc2a4* expression (63). *In silico* analysis indicated *NFKB1* and *Rela* mRNAs as target for miR-199a-5p and miR-532-3p; and despite unchanged *Nfkb1* and *Rela* mRNAs expression in muscle of diabetic rats, the p50 (NFKB1) protein content was increased, revealing a posttranscriptional regulation. Regarding that, the miR-199a-5p has already been validated as repressor of the NFKB1 protein in human peripheral blood monocytes (64). Thus, that could explain a connection between these miRNAs and the *Slc2a4*/GLUT4 and *Hk2*/HK2 expression.

Curiously, in this study, miR-93-5p was detected reduced in muscle from diabetic rats, and without correlation with GLUT4 protein. Conversely, in adipose tissue of polycystic ovary insulin resistant women, miR-93-5p was described upregulated, correlating negatively with GLUT4 protein (36). Besides, 3T3-L1 adipocytes overexpressing miR-93-5p reduced *Slc2a4* expression, whereas transfected with antisense miR-93-5p upregulated *Slc2a4* expression (36). Finally, by luciferase assay, direct binding of miR-93-5p to the *Slc2a4* 3′UTR was demonstrated, resulting in reduction of luciferase (36). Altogether, these data indicate a direct repressor effect of miR-93-5p upon *Slc2a4* mRNA, and as discussed above for miR-199a-5p and miR-532-3p, reduction in miR-93-5p should be concurring to enhance GLUT4.

In summary, the reduction of miRs-93-5p, 199a-5p, and−532-3p, which have been already demonstrated, *in vitro*, as repressors of the *Slc2a4* and/or *Hk2*, denotes that the final regulation of these targets, *in vivo*, is a balance of forces, some of them overlapping the direct expected effect of these miRNAs.

Nine miRNAs candidates to regulate *Slc2a4*/GLUT4 were observed to be unaltered in muscles of diabetic rats. Among these miRNAs, miR-106b-5p was already confirmed to target *Slc2a4* mRNA by luciferase assay in muscle L6 cells, but it was not triggered by the present DM model (46). Other miRNAs that have already been related to GLUT4 expression, such as miR-23a-3p, miR-222-3p, miR-107-3p, miR-223-3p, and miR-133a/b-3p (27), were not investigated in the present study since they were predicted to target *Slc2a4* by only a few algorithms (1–3) using the miRWalk 2.0 plataform.

As expected, STZ-induced type 1-like diabetes mellitus impaired muscle and body mass development, as well as promoted high blood glucose, plasma fructosamine and 24-h-urinary glucose excretion. Variables representative of long-term metabolic dysregulation, such as body mass, 24-h-urinary glucose excretion and plasma fructosamine, were reversed by insulin therapy, as well as *Slc2a4*/GLUT4 and *Hk2*/HK2 expression and most of the altered miRNAs were. Besides, metabolic parameters correlated positively with miR-29b-3p and miR-29c-3p and negatively with miR-199a-5p, miR-345-3p and miR-532-3p. Altogether, these data suggest that diabetes-induced impairment of glucose homeostasis and/or insulin deficiency play a fundamental role in the regulation of the altered miRNAs, which may contribute to skeletal muscle glucose disposal impairment. However, it is important to highlight that the investigation was conducted in male rats only, and that is a limitation of the present study.

In summary, we report that diabetes leads to upregulation of miR-29b-3p and miR-29c-3p expression and downregulation of miR-199a-5p and miR-532-3p expression in skeletal muscle of male rats. These miRNAs are predicted to regulate *Slc2a4*/GLUT4 and *Hk2*/HK2 expression, and their variations correlated significantly with GLUT4 and HK2 contents, which were reduced in muscle of diabetic rats, and must contribute to glucose utilization impairment. These data shed light on some miRNAs as a markers of impaired skeletal muscle glucose disposal, and, consequently, glycemic control in diabetes.

## Author contributions

JE and UM conceived and designed the experiments. JE, CY, DP-J, and FG-R performed the experimental procedures. JE and UM analyzed data. JE, FE, and UM prepared the manuscript.

### Conflict of interest statement

The authors declare that the research was conducted in the absence of any commercial or financial relationships that could be construed as a potential conflict of interest.

## References

[1] Bacha F Klinepeter Bartz S. Insulin resistance, role of metformin and other non-insulin therapies in pediatric type 1 diabetes. Pediatr Diabetes (2016) 17:545–58. 10.1111/pedi.12337 26592507

[2] DeFronzo RA Tripathy D. Skeletal muscle insulin resistance is the primary defect in type 2 diabetes. Diabetes Care (2009) 32(Suppl 2):S157–63. 10.2337/dc09-S302 19875544 PMC2811436

[3] DeFronzo RA. Pathogenesis of type 2 diabetes mellitus. Med Clin North Am. (2004) 88:787–835. 10.1016/j.mcna.2004.04.013 15308380

[4] Zierath JR Krook A Wallberg-Henriksson H. Insulin action and insulin resistance in human skeletal muscle. Diabetologia (2000) 43:821–35. 10.1007/s001250051457 10952453

[5] Klip A. The many ways to regulate glucose transporter 4. Appl Physiol Nutr Metab. (2009) 34:481–7. 10.1139/H09-047 19448718

[6] Leto D Saltiel AR. Regulation of glucose transport by insulin: traffic control of GLUT4. Nat Rev Mol Cell Biol. (2012) 13:383–96. 10.1038/nrm3351 22617471

[7] Herman MA Kahn BB. Glucose transport and sensing in the maintenance of glucose homeostasis and metabolic harmony. J Clin Invest (2006) 116:1767–75. 10.1172/JCI29027 16823474 PMC1483149

[8] Corrêa-Giannella ML Machado UF. SLC2A4gene: a promising target for pharmacogenomics of insulin resistance. Pharmacogenomics (2013) 14:847–50. 10.2217/pgs.13.45 23746177

[9] Hardin DS Dominguez JH Garvey WT. Muscle group-specific regulation of GLUT 4 glucose transporters in control, diabetic, and insulin-treated diabetic rats. Metabolism (1993) 42:1310–5. 8412743 10.1016/0026-0495(93)90130-g

[10] Machado UF Shimizu Y Saito M. Decreased glucose transporter (GLUT 4) content in insulin-sensitive tissues of obese aurothioglucose- and monosodium glutamate-treated mice. Horm Metab Res. (1993) 25:462–5. 8225198 10.1055/s-2007-1002149

[11] Okamoto MM Anhê GF Sabino-Silva R Marques MF Freitas HS Mori RC. Intensive insulin treatment induces insulin resistance in diabetic rats by impairing glucose metabolism-related mechanisms in muscle and liver. J Endocrinol. (2011) 211:55–64. 10.1530/JOE-11-0105 21746792

[12] Yonamine CY Pinheiro-Machado E Michalani ML Alves-Wagner AB Esteves JV Freitas HS. Resveratrol improves glycemic control in type 2 diabetic obese mice by regulating glucose transporter expression in skeletal muscle and liver. Molecules (2017) 22:E1180. 10.3390/molecules22071180 28708105 PMC6152102

[13] Dohm GL Elton CW Friedman JE Pilch PF Pories WJ Atkinson SM Jr. Decreased expression of glucose transporter in muscle from insulin-resistant patients. Am J Physiol. (1991) 260(3 Pt 1):E459–63. 2003599 10.1152/ajpendo.1991.260.3.E459

[14] Gaster M Staehr P Beck-Nielsen H Schrøder HD Handberg A. GLUT4 is reduced in slow muscle fibers of type 2 diabetic patients: is insulin resistance in type 2 diabetes a slow, type 1 fiber disease? Diabetes (2001) 50:1324–9. 10.2337/diabetes.50.6.1324 11375332

[15] Kampmann U Christensen B Nielsen TS Pedersen SB Ørskov L Lund S. GLUT4 and UBC9 protein expression is reduced in muscle from type 2 diabetic patients with severe insulin resistance. PLoS ONE (2011) 6:e27854. 10.1371/journal.pone.0027854 22114711 PMC3218059

[16] Zisman A Peroni OD Abel ED Michael MD Mauvais-Jarvis F Lowell BB. Targeted disruption of the glucose transporter 4 selectively in muscle causes insulin resistance and glucose intolerance. Nat Med. (2000) 6:924–8. 10.1038/78693 10932232

[17] Kim JK Zisman A Fillmore JJ Peroni OD Kotani K Perret P. Glucose toxicity and the development of diabetes in mice with muscle-specific inactivation of GLUT4. J Clin Invest. (2001) 108:153–60. 10.1172/JCI10294 11435467 PMC353719

[18] Tsao TS Burcelin R Katz EB Huang L Charron MJ. Enhanced insulin action due to targeted GLUT4 overexpression exclusively in muscle. Diabetes (1996) 45:28–36. 8522056 10.2337/diab.45.1.28

[19] Leturque A Loizeau M Vaulont S Salminen M Girard J. Improvement of insulin action in diabetic transgenic mice selectively overexpressing GLUT4 in skeletal muscle. Diabetes (1996) 45:23–7. 8522055 10.2337/diab.45.1.23

[20] Bartel DP. MicroRNAs: genomics, biogenesis, mechanism, and function. Cell (2004) 116:281–97. 10.1016/S0092-8674(04)00045-5 14744438

[21] Friedman RC Farh KK Burge CB Bartel DP. Most mammalian mRNAs are conserved targets of microRNAs. Genome Res. (2009) 19:92–105. 10.1101/gr.082701.108 18955434 PMC2612969

[22] Guay C Roggli E Nesca V Jacovetti C Regazzi R. Diabetes mellitus, a microRNA-related disease? Transl Res. (2011) 157:253–64. 10.1016/j.trsl.2011.01.009 21420036

[23] Park SY Jeong HJ Yang WM Lee W. Implications of microRNAs in the pathogenesis of diabetes. Arch Pharm Res. (2013) 36:154–66. 10.1007/s12272-013-0017-6 23361585

[24] Zhu H Shyh-Chang N Segrè AV Shinoda G Shah SP Einhorn WS. The Lin28/let-7 axis regulates glucose metabolism. Cell (2011) 147:81–94. 10.1016/j.cell.2011.08.033 21962509 PMC3353524

[25] Agarwal P Srivastava R Srivastava AK Ali S Datta M. miR-135a targets IRS2 and regulates insulin signaling and glucose uptake in the diabetic gastrocnemius skeletal muscle. Biochim Biophys Acta (2013) 1832:1294–303. 10.1016/j.bbadis.2013.03.021 23579070

[26] Bork-Jensen J Scheele C Christophersen DV Nilsson E Friedrichsen M Fernandez-Twinn DS. Glucose tolerance is associated with differential expression of microRNAs in skeletal muscle: results from studies of twins with and without type 2 diabetes. Diabetologia (2015) 58:363–73. 10.1007/s00125-014-3434-2 25403480 PMC4287682

[27] Esteves JV Enguita FJ Machado UF. MicroRNAs-mediated regulation of skeletal muscle GLUT4 expression and translocation in insulin resistance. J Diabetes Res. (2017) 2017:7267910. 10.1155/2017/7267910 28428964 PMC5385897

[28] Pinto-Junior DC Silva KS Michalani ML Yonamine CY Esteves JV Fabre NT. Advanced glycation end products-induced insulin resistance involves repression of skeletal muscle GLUT4 expression. Sci Rep. (2018) 8:8109. 10.1038/s41598-018-26482-6 29802324 PMC5970140

[29] Thacker JS Yeung DH Staines WR Mielke JG. Total protein or high-abundance protein: Which offers the best loading control for Western blotting? Anal Biochem. (2016) 496:76–8. 10.1016/j.ab.2015.11.022 26706797

[30] Yonamine CY Pinheiro-Machado E Michalani ML Freitas HS Okamoto MM Corrêa-Giannella ML. Resveratrol improves glycemic control in insulin-treated diabetic rats: participation of the hepatic territory. Nutr Metab (Lond.). (2016) 13:44. 10.1186/s12986-016-0103-0 27366200 PMC4928352

[31] Livak KJ Schmittgen TD. Analysis of relative gene expression data using real-time quantitative PCR and the 2^−Δ*ΔCT*^ Method. Methods (2001) 25:402–8. 10.1006/meth.2001.1262 11846609

[32] Gerlinger-Romero F Yonamine CY Junior DC Esteves JV Machado UF. Dysregulation between TRIM63/FBXO32 expression and soleus muscle wasting in diabetic rats: potential role of miR-1-3p,−29a/b-3p, and−133a/b-3p. Mol Cell Biochem. (2017) 427:187–99. 10.1007/s11010-016-2910-z 28000044

[33] Dweep H Gretz N. miRWalk2.0: a comprehensive atlas of microRNA-target interactions. Nat Methods (2015) 12:697. 10.1038/nmeth.3485 26226356

[34] He A Zhu L Gupta N Chang Y Fang F. Overexpression of micro ribonucleic acid 29, highly up-regulated in diabetic rats, leads to insulin resistance in 3T3-L1 adipocytes. Mol Endocrinol. (2007) 21:2785–94. 10.1210/me.2007-0167 17652184

[35] Massart J Sjögren RJO Lundell LS Mudry JM Franck N O'Gorman DJ. Altered miR-29 expression in type 2 diabetes influences glucose and lipid metabolism in skeletal muscle. Diabetes (2017) 66:1807–18. 10.2337/db17-0141 28404597

[36] Chen YH Heneidi S Lee JM Layman LC Stepp DW Gamboa GM. miRNA-93 inhibits GLUT4 and is overexpressed in adipose tissue of polycystic ovary syndrome patients and women with insulin resistance. Diabetes (2013) 62:2278–86. 10.2337/db12-0963 23493574 PMC3712080

[37] Chavali V Tyagi SC Mishra PK. Differential expression of dicer, miRNAs, and inflammatory markers in diabetic Ins2+/- Akita hearts. Cell Biochem Biophys. (2014) 68:25–35. 10.1007/s12013-013-9679-4 23797610 PMC4085798

[38] Punga T Le Panse R Andersson M Truffault F Berrih-Aknin S Punga AR. Circulating miRNAs in myasthenia gravis: miR-150-5p as a new potential biomarker. Ann Clin Transl Neurol. (2014) 1:49–58. 10.1002/acn3.24 25356381 PMC4207504

[39] Yan ST Li CL Tian H Li J Pei Y Liu Y. MiR-199a is overexpressed in plasma of type 2 diabetes patients which contributes to type 2 diabetes by targeting GLUT4. Mol Cell Biochem. (2014) 397:45–51. 10.1007/s11010-014-2170-8 25084986

[40] Barsanti C Trivella MG D'Aurizio R El Baroudi M Baumgart M Groth M. Differential regulation of microRNAs in end-stage failing hearts is associated with left ventricular assist device unloading. Biomed Res Int. (2015) 2015:592512. 10.1155/2015/592512 25710008 PMC4330954

[41] Chen L Chen Y Zhang S Ye L Cui J Sun Q. MiR-540 as a novel adipogenic inhibitor impairs adipogenesis via suppression of PPARγ. J Cell Biochem. (2015) 116:969–76. 10.1002/jcb.25050 25560764

[42] Lee KP Shin YJ Panda AC Abdelmohsen K Kim JY Lee SM. miR-431 promotes differentiation and regeneration of old skeletal muscle by targeting Smad4. Genes Dev (2015) 29:1605–17. 10.1101/gad.263574.115 26215566 PMC4536309

[43] Wang JX Zhang XJ Feng C Sun T Wang K Wang Y. MicroRNA-532-3p regulates mitochondrial fission through targeting apoptosis repressor with caspase recruitment domain in doxorubicin cardiotoxicity. Cell Death Dis. (2015) 6:e1677. 10.1038/cddis.2015.41 25766316 PMC4385919

[44] Kushwaha P Khedgikar V Sharma D Yuen T Gautam J Ahmad N. MicroRNA 874-3p exerts skeletal anabolic effects epigenetically during weaning by suppressing Hdac1 expression. J Biol Chem. (2016) 291:3959–66. 10.1074/jbc.M115.687152 26663087 PMC4759174

[45] Wang KJ Zhao X Liu YZ Zeng QT Mao XB Li SN. Circulating MiR-19b-3p, MiR-134-5p and MiR-186-5p are promising novel biomarkers for early diagnosis of acute myocardial infarction. Cell Physiol Biochem. (2016) 38:1015–29. 10.1159/000443053 26939053

[46] Zhou T Zhou T Meng X Che H Shen N Xiao D Song X. regulation of insulin resistance by multiple MiRNAs via targeting the GLUT4 signalling pathway. Cell Physiol Biochem. (2016) 38:2063–78. 10.1159/000445565 27165190

[47] Zhu XD Chi JY Liang HH Huangfu LT Guo ZD Zou H. MicroRNA-377 mediates cardiomyocyte apoptosis induced by cyclosporin A. Can J Cardiol. (2016) 32:1249–59. 10.1016/j.cjca.2015.11.012 26948033

[48] Wallberg-Henriksson H Zierath JR. GLUT4: a key player regulating glucose homeostasis? Insights from transgenic and knockout mice (review). Mol Membr Biol. (2001) 18:205–11. 10.1080/09687680110072131 11681787

[49] Krook A. A balancing act of optimising insulin dose and insulin sensitivity in type 1 diabetes. J Endocrinol. (2011) 211:1–2. 10.1530/JOE-11-0263 21824897

[50] Patel OV Casey T Dover H Plaut K. Homeorhetic adaptation to lactation: comparative transcriptome analysis of mammary, liver, and adipose tissue during the transition from pregnancy to lactation in rats. Funct Integr Genomics (2011) 11:193–202. 10.1007/s10142-010-0193-0 20852911

[51] Ruan H Zarnowski MJ Cushman SW Lodish HF. Standard isolation of primary adipose cells from mouse epididymal fat pads induces inflammatory mediators and down-regulates adipocyte genes. J Biol Chem. (2003) 278:47585–93. 10.1074/jbc.M305257200 12975378

[52] Kahn BB Rosen AS Bak JF Andersen PH Damsbo P Lund S. Expression of GLUT1 and GLUT4 glucose transporters in skeletal muscle of humans with insulin-dependent diabetes mellitus: regulatory effects of metabolic factors. J Clin Endocrinol Metab. (1992) 74:1101–9. 1569156 10.1210/jcem.74.5.1569156

[53] Klip A Tsakiridis T Marette A Ortiz PA. Regulation of expression of glucose transporters by glucose: a review of studies *in vivo* and in cell cultures. FASEB J. (1994) 8:43–53. 8299889 10.1096/fasebj.8.1.8299889

[54] Neufer PD Carey JO Dohm GL. Transcriptional regulation of the gene for glucose transporter GLUT4 in skeletal muscle. Effects of diabetes and fasting. J Biol Chem. (1993) 268:13824–9. 7686145

[55] Hager SR Pastorek D Jochen AL Meier D. Divergence between GLUT4 mRNA and protein abundance in skeletal muscle of insulin resistant rats. Biochem Biophys Res Commun. (1991) 181:240–5. 1958193 10.1016/s0006-291x(05)81408-1

[56] Seraphim PM Nunes MT Giannocco G Machado UF. Age related obesity-induced shortening of GLUT4 mRNA poly(A) tail length in rat gastrocnemius skeletal muscle. Mol Cell Endocrinol. (2007) 276:80–7. 10.1016/j.mce.2007.07.004 17709177

[57] Zhou Y Gu P Shi W Li J Hao Q Cao X. MicroRNA-29a induces insulin resistance by targeting PPARδ in skeletal muscle cells. Int J Mol Med. (2016) 37:931–8. 10.3892/ijmm.2016.2499 26936652 PMC4790643

[58] Guedes EC França GS Lino CA Koyama FC Moreira Ldo N Alexandre JG. MicroRNA expression signature is altered in the cardiac remodeling induced by high fat diets. J Cell Physiol. (2016) 231:1771–83. 10.1002/jcp.25280 26638879

[59] Song H Ding L Zhang S Wang W. MiR-29 family members interact with SPARC to regulate glucose metabolism. MiR-29 family members interact with SPARC to regulate glucose metabolism. Biochem Biophys Res Commun. (2018) 497:667–674. 10.1016/j.bbrc.2018.02.129 29462611

[60] Guo W Qiu Z Wang Z Wang Q Tan N Chen T. MiR-199a-5p is negatively associated with malignancies and regulates glycolysis and lactate production by targeting hexokinase 2 in liver cancer. Hepatology (2015) 62:1132–44. 10.1002/hep.27929 26054020

[61] Zhou Y Zheng X Lu J Chen W Li X Zhao L. Ginsenoside 20(S)-Rg3 inhibits the warburg effect via modulating DNMT3A/ MiR-532-3p/HK2 pathway in ovarian cancer cells. Cell Physiol Biochem. (2018) 45:2548–2559. 10.1159/000488273 29558748

[62] Valinezhad Orang A Safaralizadeh R Kazemzadeh-Bavili M. Mechanisms of miRNA-mediated gene regulation from common downregulation to mRNA-specific upregulation. Int J Genomics (2014) 2014:970607. 10.1155/2014/970607 25180174 PMC4142390

[63] Furuya DT Neri EA Poletto AC Anhê GF Freitas HS Campello RS. Identification of nuclear factor-κB sites in the Slc2a4 gene promoter. Mol Cell Endocrinol. (2013) 370:87–95. 10.1016/j.mce.2013.01.019 23462193

[64] Hassan T Carroll TP Buckley PG Cummins R O'Neill SJ McElvaney NG. miR-199a-5p silencing regulates the unfolded protein response in chronic obstructive pulmonary disease and α1-antitrypsin deficiency. Am J Respir Crit Care Med. (2014) 189:263–73. 10.1164/rccm.201306-1151OC 24299514

